# Origins of Electromechanical Behavior in Surface-Localized Nanocomposites: Insights into Crack Network Dynamics and Particle Network Rearrangements

**DOI:** 10.1021/acsapm.5c01519

**Published:** 2025-07-10

**Authors:** Emily A. Ryan, Natalie E. Raia, John R. Reynolds, Meisha L. Shofner

**Affiliations:** † School of Materials Science and Engineering, 1372 Georgia Institute of Technology, Atlanta, Georgia 30322, United States; ‡ School of Chemistry and Biochemistry, Georgia Institute of Technology, Atlanta, Georgia 30322, United States

**Keywords:** surface-localized nanocomposite, reduced graphene oxide, piezoresistivity, electromechanical behavior, space materials

## Abstract

There is a critical need for new technologies to support lunar and Martian exploration efforts, particularly for flexible, durable, and environmentally stable materials that can weather challenging space conditions. Electrically conductive thin films are critical for numerous applications, including structural health monitoring, charge dissipation, micrometeoroid and orbital debris impact detection, and electrodynamic dust shielding. Surface-localized nanocomposites (SLNCs) offer a promising alternative to metallic and ceramic films as flexible, durable, thin film conductors. This study examines the electromechanical properties in tension and bending of SLNCs produced via melt infiltration with chemically modified reduced graphene oxide (CMrGO) as the conductive component with three polymer materials which serve as both the substrate and the composite matrix in the SLNC: olefin block copolymer (OBC), high-density polyethylene (HDPE), and poly­(vinylidene fluoride) (PVDF). Monotonic electromechanical tensile testing revealed a substrate independent, linear piezoresistive response in the elastic regime and highly substrate dependent nonlinear piezoresistive response beyond yield. Substrate ductility strongly affected the measurable gauge factor (GF) in these systems, with OBC and HDPE SLNCs having GFs of 20–100, compared to only 2 for PVDF. Static bending tests showed small changes in resistance even at sharp bending radii. The low GF in the elastic regime and at sharp bending radii is beneficial for both active and passive electronic applications for inflatable structures, drapeable covers, and flexible robotics. In situ microscopy during uniaxial tensile deformation identified crack network development as a key mechanism contributing to the nonlinear piezoresistive response after yield. Optical profilometry revealed the formation of partial thickness cracks at low strains which grew deeper and wider with increasing strain. Finally, cyclic testing indicated that in addition to crack formation and closure, particle network rearrangement associated with matrix relaxation plays a significant role in the piezoresistive response, particularly in the elastic regime.

## Graphical abstract

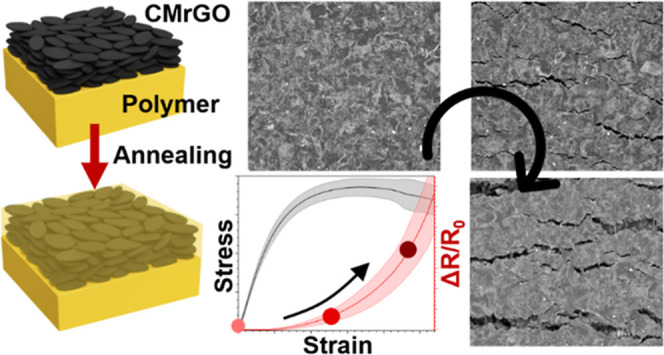

## 1 Introduction

Future space exploration missions will include a variety of new applications for polymer film materials that were not encountered during prior lunar exploration and low Earth orbit (LEO) missions.^1^ For example, NASA’s Artemis Lunar Base Camp, targeted to be established by 2030, will include an inflatable crew habitat constructed from flexible fabric and film materials, while the Orbital Reef, a privatized LEO research vessel, will be equipped with an inflatable habitat module for a crew of up to ten astronauts.^2,3^ Beyond inflatable structures, polymer films will be applied to flexible robotics, drapeable covers, repair appliques for flexible surfaces, and protective shields for rovers and equipment.^4,5^ Integrating electrical conductivity into these polymer films could enable additional active and passive capabilities, such as micrometeoroid impact detection,^6^ strain mapping for inflatable structures and fiber reinforced composites,^7^ electrostatic charge dissipation (ESD),^8,9^ electromagnetic interference shielding, and electrodynamic dust mitigation.^10^ One challenge in material development for these applications is the fabrication of large-area, thin film conductors with sufficient mechanical and environmental robustness for challenging space environments.^11^ Flexible polymer film conductors must be sufficiently flexible to be folded during stowage and deployment, potentially withstand repeated actuation during use, and remain stable in the harsh space environment, including exposure to vacuum, UV, thermal effects, high energy radiation, and abrasive lunar and Martian dust during planetary deployments.^1,12^

Historically, metallized polymer films, deposited via vapor deposition and sputtering techniques, have been widely applied for thermal control and ESD protective surfaces on spacecraft and ground vehicles.^13^ More recently, ceramic oxide films such as indium tin oxide (ITO), have also been implemented for ESD protection in transparent film applications and thermal control surfaces, though crack formation in these layers is an ongoing challenge in spacecraft construction and deployment.^14–16^ Additionally, space radiation has also been shown to cause embrittlement and delamination of metallic thin film coatings, leading to the formation of large cracks and disruption of electrical conductivity even in low strain configurations.^17^ While improvements in metallic film adhesion to a variety of polymer substrates, as well as strategies to mitigate strain effects via substrate prestretching, interconnect design, and conductor trace design have allowed for significant advancement of metal-on-polymer stretchable electronics, future space deployments would benefit from expanded options for conductive polymer thin film materials.^18–21^

Nanocomposite-based conductive thin films have shown significant promise as durable, conductive surfaces due to their high flexibility and ease of fabrication.^1^ Nanocomposite films can be formed via solution casting from polymer–particle mixtures such as latexes,^7^ network polymer precursors,^22^ or dissolved polymer solutions, and have been demonstrated in self-sensing and flexible conductor applications.^23,24^ In solution-based nanoparticle film deposition approaches, poor particle dispersion, uneven deposition, and poor substrate adhesion can all reduce the electromechanical durability of these flexible conductors.^24,25^ In addition to solution-processed films, researchers have also investigated the formation of conductive nanocomposites via infiltration of percolated nanoparticle packings with prepolymer and in situ particle growth from deposited monomer-precursor films to produce highly stretchable conductive substrates.^26–28^

Melt infiltration is an alternative technique to ink deposition, liquid infiltration, and in situ particle generation, which leverages the capillary infiltration of percolated nanoparticle packings with thermoplastic polymer to produce nanocomposite films.^29^ Our prior work on melt infiltration of chemically modified reduced graphene oxide (CMrGO) nanoparticle films has shown that melt infiltration can be used to produce thick (∼20 μm), highly loaded nanocomposite films on a variety of thermoplastic substrates.^30^ These nanocomposite films are well-adhered to the polymer substrate via entangled polymer chains between the substrate and nanocomposite layer.^30^ This infiltration-based method prevents film delamination which is known to cause early failure in both metallic and nanocomposite conductive films on compliant substrates.^18,23,28^ The infiltrated nanocomposite film structure is referred to as a surface-localized nanocomposite (SLNC) in this work, and in our prior work, to indicate that the nanocomposite region is only present at the film surface and not the entire cross-section of the film. This term also differentiates these structures from solution-deposited films produced by ink deposition.

In addition to producing an integrated nanocomposite surface, melt infiltration has also been shown to preserve the initial conductive particle network arrangement.^31^ This work leveraged this feature to directly compare the effects of matrix ductility on the electromechanical response of infiltrated nanocomposite films across three thermoplastic substrates: olefin block copolymer (OBC), high density polyethylene (HDPE), and polyvinylidene fluoride (PVDF). Prior research efforts in the area of infiltrated nanocomposite structures have not explored the electromechanical response of this class of materials which is critical to the durability and performance of these surfaces as functional materials for electrical applications.^29^ CMrGO, a functionalized version of reduced graphene oxide (rGO) was chosen as the conductive particle due to rGO’s stability in the space environment including UV, high energy radiation, and high vacuum exposure.^14,28^

To measure and understand the origin of electromechanical behavior in these infiltrated nanocomposite films, a multistage characterization approach was used. Under monotonic strain conditions all three substrates, OBC, HDPE, and PVDF, showed linear electromechanical responses in the elastic regime, and nonlinear, substrate dependent responses beyond yield. Under static bending, minimal increases in resistance (<20%) where measured for bending radii larger than 2 mm, which suggested suitability for flexible, foldable materials in sensing applications, as passive discharge materials, and as active electronic elements. In situ SEM, optical microscopy, and profilometry linked the formation and evolution of crack networks during plastic deformation to the nonlinear electromechanical response, consistent with the mechanism observed in other stiff, conductive films on compliant substrates. Cyclic tension testing highlighted the contributions from particle network reconfiguration, crack formation, and crack closure on cycle-dependent electromechanical responses under elastic and plastic strains. These insights into the underlying mechanisms and overall electromechanical stability and responsiveness of SLNCs can be used to guide selection of these materials for active and passive applications in space exploration.

## 2 Materials and Methods

### 2.1 Materials

rGO nanoplatelets were obtained from ACS Materials and had a reported monolayer diameter of 0.5–10 μm. CMrGO was prepared using the purchased nanoplatelets according to Seibers et al.^32^ Full details can be found in the Supporting Information. HDPE films with a nominal thickness of 101 μm (McMaster-Carr, 1939T42) and PVDF films with a nominal thickness 76 μm (CS Hyde, PVDF/Kynar 720 Resin, 32-3F-26) were used as substrates and used as received.^32^ OBC substrates were produced by melt pressing segments of OBC filament (3DXTech, Evolv3D OBC, blue) into 2 mm thick plaques at 210 °C for 20 min under 2.55 MPa of pressure in a walled mold with a plunger top. Films were pressed from 1 cm × 1 cm plaques into 75 μm thick films under the same temperature and pressure conditions between two aluminum plates spaced by 75 μm shims to control thickness. Two 50 × 70 mm films were extracted from the interior of each pressed film to remove edges which had slight variations in thickness.

### 2.2 Fabrication of SLNCs

SLNCs were produced according to methods similar to those described in our previous work.^30^ In short, CMrGO particles were dispersed by sonication (130 W, 40 kHz, Fisher Scientific, FS30H) in chloroform (Fisher Scientific, AA32614K7) at a concentration of 10 mg/mL. The dispersed particle solutions were sprayed onto the polymer film substrates using a commercial airbrush (Iwata, Eclipse HP CS Dual Action Airbrush) with N_2_ gas as the carrier at 10 bar. The layer was deposited using multiple passes to produce uniform, opaque particle films with a total areal density of 1 mg/cm^2^. Each sprayed particle film was then processed by compression molding at a temperature which corresponds to 99.5% melt conversion and 2.55 MPa of pressure to achieve complete infiltration of the particle layer resulting in a bilayer film where the top 20 μm is SLNC and the bottom 60–65 μm in PVDF and OBC and 80–85 μm in HDPE is neat polymer substrate (Figure S1). The 99.5% melt conversion temperatures for each substrate were calculated from differential scanning calorimetry (DSC) thermograms collected at 10 °C/min (TA Instruments, Discovery DSC). Complete processing details, including determination of 99.5% melt conversion and mold configuration, are provided in the Supporting Information.

### 2.3 Characterization

Electrical measurements of the films were made using a Keithley 2400 SourceMeter and a four-point line probe (Signatone, SP4-62085TFJ). Each SLNC sample was measured in five locations each on two films processed separately. To provide a reference value for the CMrGO particle network without polymer infiltration, a sprayed particle film of the same areal density was subjected to the same compression conditions at room temperature and measured in the same way on a PVDF substrate.

### 2.4 Electromechanical Property Measurements

An Instron 68TM-R5566 frame, 100 N universal load cell, and micro tensile grips were used to test the samples to collect electromechanical properties. An ASTM D638 Type V tensile dog bone sample geometry was used for electromechanical testing to ensure strain occurred over a known gauge length for all samples.^33^ Two-point resistance values were measured at a frequency of 10 Hz using a digital multimeter (Digilent, Analog Discovery Pro 5250) and the Waveforms recording software. Electrical contact was made by applying a strip of Ag ink (Ted Pella Inc., Electrodag 1415M) just outside the gauge region. Once dry, a tab of copper tape was applied to the Ag contact and connected to the multimeter via a 100 AWG insulated wire. The region with the copper tab was fitted into the grip of the tensile frame and electrically isolated by 320 grit sandpaper which also provided improved grip. For tests to failure, samples were elongated at testing speeds of 0.5 mm/min (5%/min) for PVDF and 2.5 mm/min (26%/min) for HDPE and OBC specimens. Tensile strain rates were calculated using the crosshead displacement rate and the original gauge length of the specimen. Strain values are reported as the percentage change in sample length, as measured by crosshead displacement versus the original gauge length of the specimen.

The normalized change in resistance (Δ*R*/*R*_0_) value was calculated using eq 1

1 $$\frac{\Delta R}{R_{0}}=\frac{R-R_{0}}{R_{0}}$$

where *R*_0_ is the average resistance measured with the sample in the tensile configuration at 0% strain directly before the test, or with the sample placed flat on a surface for the bending configuration, and *R* is the single point resistance measurement at each strain value.

For cyclic tests, the same tensile sample geometry, strain rates, and electrical contact methods were used. The amplitude for all tests was 2% strain, and the frequency was set by the applied strain rate. For all three materials, one test was performed in the elastic deformation region (4–6% strain for OBC, 2–4% strain for HDPE and PVDF) and one test was performed in the plastic deformation region (20–22% strain for OBC, 13–15% strain for HDPE, 8–10% strain for PVDF). Prior to each test, the samples were strained to the lower strain cycle value and held for 10 s. The resistance at this plateau was used as *R*_0_ in eq 1, in place of the unstretched resistance value used in the quasi-static and bending tests, to better demonstrate the evolution of the electromechanical response under cyclic strain.

Static bending tests were performed using 6 mm wide by 50 mm long rectangular specimens with Ag ink and copper tape contacts as above. For each bending radius, the sample was wrapped around the corresponding radius step on a set of custom bending jigs with fixed radii ranging from 20 mm to 1 mm. The bending jigs were produced by material extrusion additive manufacturing using a Stratasys F370 printer and acrylonitrile butadiene styrene filament. A GitHub repository containing the STL files of the bending jigs are included with the Supporting Information files.

### 2.5 In Situ Optical Profilometry and SEM

In situ scanning electron microscopy and profilometry images were collected of samples under strain using a custom microtensile frame produced by material extrusion additive manufacturing with a Bambu Lab P1S printer and polylactic acid filament. A GitHub repository containing the STL files and assembly instructions for this microtensile fixture are included in the Supporting Information. The microtensile frame was capable of strain adjustment steps of 1.1% strain for a nominal gauge length of 9.53 mm corresponding to ASTM D638 Type V tensile specimens. The strain was manually set via a small set knob, and the tensile frame containing the samples was either placed in the SEM chamber or on the optical microscope stage for image collection. Corresponding electromechanical data was collected in the same manner as described in Section 2.4, but no sandpaper was used in the grip portion of the microtensile frame as the structure is not conductive.

SEM images were collected using a Phenom XL G2 desktop SEM which has a large chamber volume which can easily accommodate the microtensile fixture. Backscatter and secondary electron images were collected at a beam intensity of 10 keV in the “high” vacuum condition (0.1 Pa). High magnification images were collected at 2000×, and low magnification images were collected at 500×. Specimens were imaged with no additional conductive coating as SLNCs remain sufficiently conductive even at high strains to produce clear images in SEM.

For optical profilometry, a Keyence VHX-7000 microscope with a VHX-E100 objective was used. The optical profiles were produced using the Z-stack reconstruction tool and measured using the Keyence imaging software. Line cut data was also generated via the Keyence imaging software.

## 3 Results and Discussion

### 3.1 Surface-Localized Nanocomposite in Semicrystalline Matrices

The fabrication process of the melt infiltrated nanocomposite films is shown in Figure 1. A thick layer of randomly oriented CMrGO particles was deposited on the surface of the target thermoplastic substrate via spraying at an areal density of 1 mg/cm^2^, and then the sprayed substrate was subjected to a 30 min annealing process to infiltrate the substrate into the nanocomposite system. Since the CMrGO particles do not migrate during infiltration, this annealing process resulted in a surface-localized nanocomposite film which was well-integrated with the thermoplastic substrate via an entangled polymer network which spanned from the substrate to the nanocomposite layer.^30,31^

**Figure 1:**
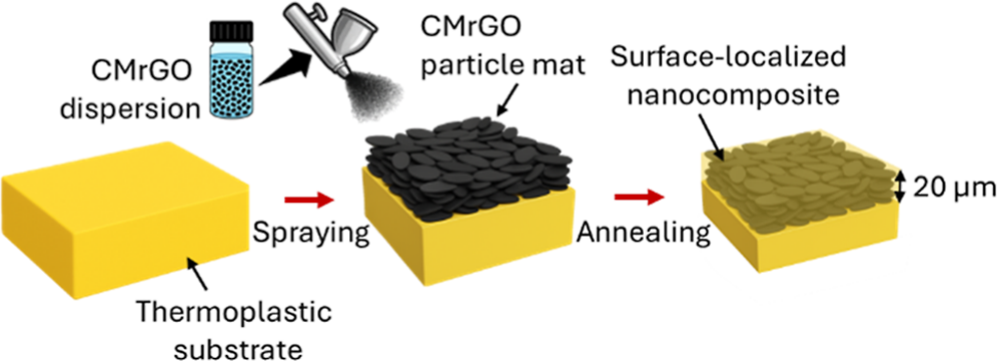
Schematic illustration of the fabrication process of a surface-localized nanocomposite film.

To produce SLNCs in these materials, each substrate plus sprayed particle film pair were subjected to processing temperatures near the end of their melting region as measured in a typical DSC experiment which has been shown to produce fully dense, infiltrated nanocomposite films.^30^ Processing temperatures were set at 99.5% melt conversion for each substrate. The DSC thermograms and tabulated values used for processing each substrate are presented in Figure S2 and Table S1, respectively. These infiltration conditions resulted in smooth, glossy surfaces with similar sheet resistances as the compressed CMrGO particle layer without polymer infiltration. A mean value and one standard deviation in sheet resistance for each SLNC are reported in Table S2. The sheet resistances measured for these materials, in the range of 200–500 ohm/sq., are more than sufficient for ESD protection and are also suitable for active applications such as electrodynamic dust shields, strain mapping, or impact detection in flexible structures.^1,6,10^

To investigate the influence of substrate ductility on the electromechanical behavior of SLNC films, three semicrystalline thermoplastic substrates with a range of elastic moduli and ductility were selected for this study. OBC had the highest ductility, with strain at break exceeding 1000%, and the lowest modulus. Its compliance was most comparable to that of polydimethylsiloxane systems frequently used as the compliant substrate in flexible and stretchable electronic devices.^34^ PVDF had the lowest ductility, with a strain at break of 15–20%, and the highest modulus. HDPE represented an intermediate case, with high ductility (strain at break >500%), and an intermediate modulus.

The range of mechanical properties, combined with the formation of similar particle networks in all three SLNCs, enabled the systematic investigation of ductility effects on the electromechanical behavior by excluding differences in particle network structure and dispersion that can occur with other processing methods such as ink deposition with different particle–matrix pairs. Comparable studies in Ag ink systems required changing both ink formulation and ink deposition parameters to ensure good adhesion in various nanocomposite-substrate pairs.^23,24^ The current study, in which the substrate polymer and the composite matrix polymer are the same material and the particle network structure is maintained, would be infeasible using a nanocomposite ink approach due to differences in dispersibility of the nanoparticles in appropriate solvents for target polymers, polymer–particle interactions in solution, and variable drying behaviors which would produce variations in the percolated particle network and adhesion at the film–substrate interface. Additionally, the infiltration process inherently produced good adhesion of the SLNC layer making delamination between the two layers unlikely and eliminating a common failure mode of metallic and ceramic thin films.^15,18^

### 3.2 Monotonic Tensile and Bending Electromechanical Results

The electromechanical response to monotonic strain was measured in each substrate to understand the stability of conductivity during tensile stress, as well the piezoresistive response of the SLNC structure under both elastic and plastic deformation. The average electromechanical response, reported as normalized change in resistance (Δ*R*/*R*_0_) according to eq 1, as a function of monotonic strain for each SLNC system is shown in Figure 2. The average mechanical response (±1σ shaded) is presented in black, while the average electromechanical response (±1σ shaded) is shown in red. Regardless of substrate stiffness, all three systems (HDPE, OBC, and PVDF) exhibited a similar trend in electromechanical behavior across a strain range extending to measured resistance values of 2 MΩ (upper measurement limit). At strains in the elastic regime of each substrate, a gradual increase in normalized resistance change was observed, followed by a more rapid increase at strain values beyond the substrate’s yield point.

**Figure 2:**
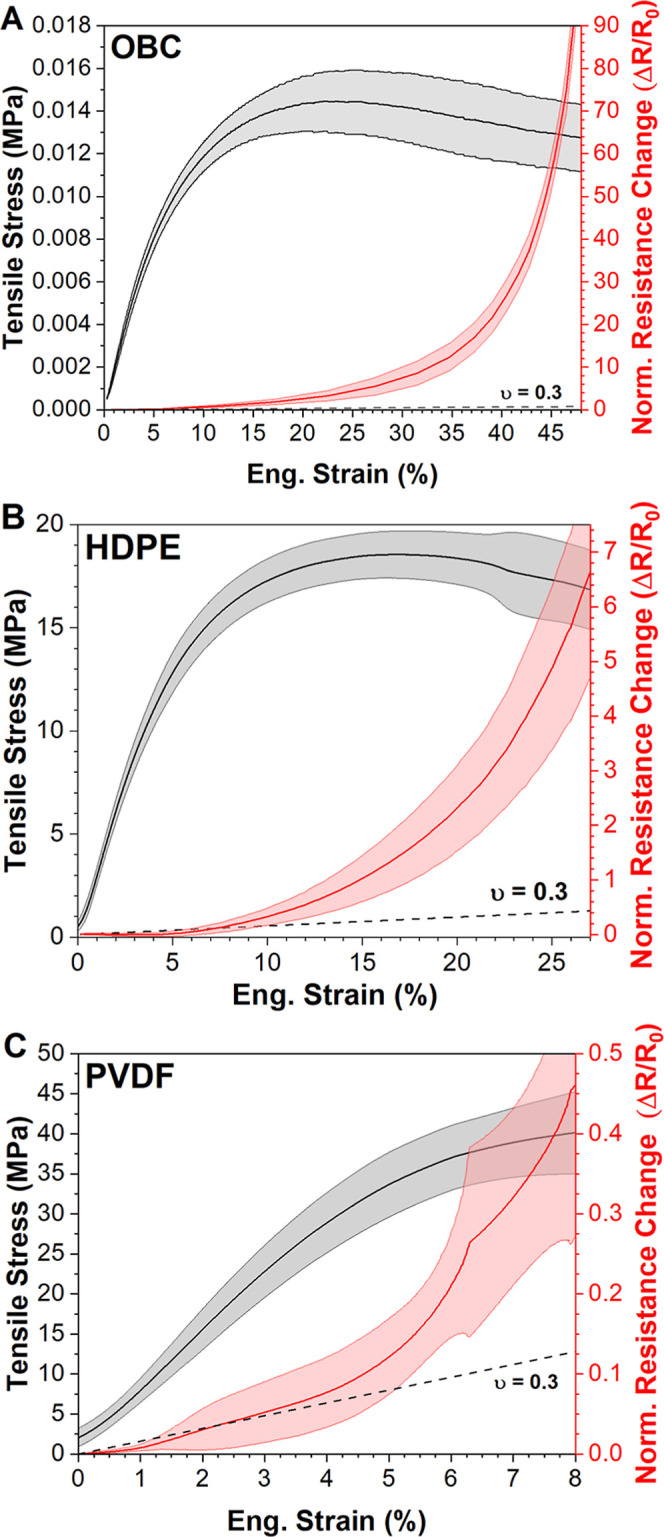
Electromechanical response of SLNCs under monotonic strain for the three substrate materials. In each panel, the shaded regions indicate ±1σ for the mechanical (black) and electromechanical (red) responses. (A) OBC SLNCs exhibited a gradual increase in Δ*R*/*R*_0_ at elastic strains, followed by a faster increase at plastic strains. (B) HDPE and (C) PVDF SLNCs showed a similar overall to OBC SLNCs.

The electromechanical behavior of a piezoresistive material can be modeled by eq 2 where ν is Poisson’s ratio of the conductor, ε is the applied strain, and Δρ/ρ_0_ is the normalized change in resistivity (ρ) of the conductor.

2 $$\frac{\Delta R}{R_{0}}=\frac{R-R_{0}}{R_{0}}=\left(1+2\nu \right)\varepsilon +\frac{\Delta \rho }{\rho_{0}}$$

The first term in eq 2 captures the contribution of Poisson’s thinning, transverse material contraction due to longitudinal stretching, which reduces the conductive cross-section and results in a normalized resistance change as a linear function of strain. This portion of the response is often the dominate mechanism in linear piezoresistive systems at strain regimes where additional mechanics which change resistivity, such as defect generation, are not present.^18^ The second term (Δρ/ρ_0_) captures the effect of all mechanisms which alter the resistivity (ρ) of the conductor.

The dashed line in each panel of Figure 2 represents the response for a conductor with a Poisson’s ratio of 0.3 and is included to highlight the divergence from linear piezoresistive behavior beyond yield in all three systems. A reproduction of Figure 2 with alternative Poisson’s ratios (ν = 0.1 and 0.5) is included in Figure S3. In the elastic regime (0–2%) for OBC and PVDF systems (Figure 2A,C), the piezoresistive response is linear and of a similar magnitude to the predicted response indicating that for monotonic testing in this strain range the piezoresistive response was due primarily to Poisson’s thinning in these materials. Interestingly, in the elastic regime of HDPE (Figures 2B and S3B), the piezoresistive response fell below the expected linear response. Reduced resistivity (Δρ/ρ_0_ < 0) has been observed in percolated particle systems which undergo network reconfiguration resulting in increased particle–particle connectivity and in metallic thin film systems which experience grain growth during cyclic fatigue testing.^35–37^

All three systems begin to show nonlinear increases in normalized resistance near yield, as indicated by divergence from the model (dashed, solid black lines, Figure S3). This nonlinearity is attributed to mechanisms which alter the apparent resistivity of the SLNC. In percolated nanocomposite systems, three mechanisms have been observed to account for nonlinearity in the piezoresistive response. At the shortest length scale, deformation of conjugated carbon nanostructures can result in changes in particle-level conductivity, though prior work on particle-only graphene films indicated that this is not a primary contribution to the nonlinear piezoresistive behavior.^35,38^ Changes in the percolated network structure/interparticle spacing and the generation of macroscopic defects, such as cracks and voids, are more commonly identified as the sources of nonlinear piezoresistivity in nanocomposite systems.^35,36^ The remainder of this study will focus on identification of the dominant underlying mechanisms which generate nonlinear piezoresistivity in this alternatively processed nanocomposite film structure.

One other notable difference between these systems is the magnitude of the electromechanical response, as indicated by the maximum value on the Y2-axis, prior to electrical or mechanical failure of the specimens. In the OBC SLNCs, stable increases in resistance up to 100× (Δ*R*/*R*_0_ > 100) at 50% strain were observed resulting in a gauge factor ((Δ*R*/*R*_0_)/ε) of 200 without mechanical failure or electrical failure of the specimen. The high extensibility of the OBC substrate allowed the substrate to support wide crack openings without mechanical failure. In this system, electrical failure in the SLNC layer occurred well before physical fracture of the specimen (50–60% and 400–1000%, respectively). This is similar to the behavior observed in particle mats embedded in elastomeric substrates and stretchable Ag inks on elastomeric substrates, where electrical disconnection occurs before complete specimen failure.^23,26,27^

In contrast, PVDF SLNCs exhibited only small changes in normalized resistance (Δ*R*/*R*_0_ < 1) before coupled electromechanical and mechanical failure at approximately 10% strain. Despite these overall small changes in resistance, an average gauge factor of 5.6 is measured at 8% strain. Coupled film–substrate failure, as seen in the PVDF SLNC system, is observed in metallic thin films on compliant substrates when the film is well-adhered and the substrate has low ductility, resulting in rapid crack propagation from the conductive layer through the thin film substrate.^18^ HDPE SLNCs represented an intermediate case, where stable resistance changes of 20–100× were observed prior to rapid increases in resistance and subsequent electrical failure around 30% strain for most specimens. At 27% strain, HDPE specimens attained an average gauge factor of 24. Similar to OBC, mechanical failure of the HDPE SLNC samples occurred at higher strains (200–500%) than electrical failure (32–34%). The ability to support large plastic deformations and produce a large gauge factor could be advantageous for detecting the occurrence and extent of damage in sudden damage events, such as micrometeoroid impacts and puncture events which are critically important to the structural health of inflatable space structures.^6^

In addition to the tensile strain response, the piezoresistive behavior of these materials under bending was examined. Many space applications where flexible films would be advantageous are expected to involve repeated bending events including manipulation of flexible robotic elements, as well as stowage and use of drapeable covers for equipment and rovers.^4,5^ In other applications, such as inflatable habitats, materials will be folded for transport and unfolded for use which will require stable electrical properties during single, or few, bend events. To assess the piezoresistive bending response, a custom-designed set of bend fixtures, with bend radii ranging from 20 mm to 1 mm, was used to take two-point resistance measurements of specimens under static bending conditions (Figure 3A). Results were produced for both exterior and interior SLNC orientations to probe tensile and compressive strains, respectively.

**Figure 3:**
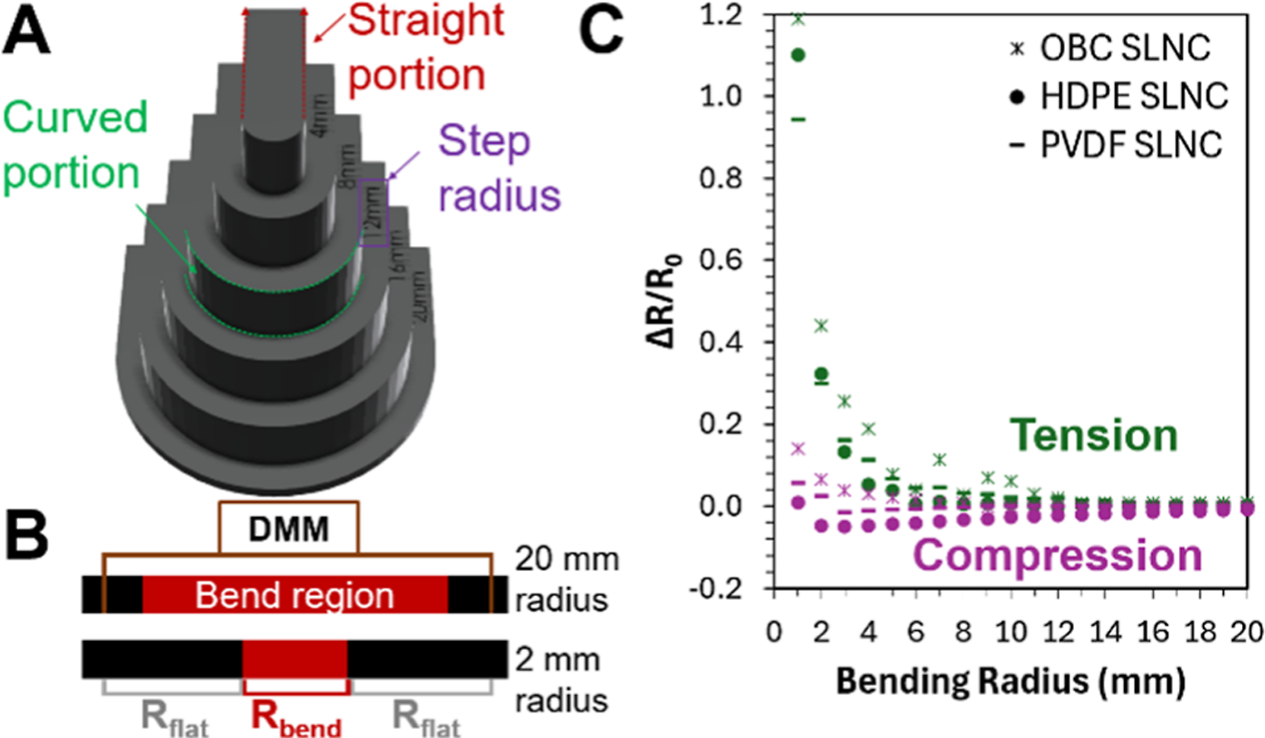
(A) Custom bend fixture used for static bending experiments. (B) Schematic showing the geometry consideration used for correction of measured normalized resistance change. (C) Normalized resistance change (Δ*R*/*R*_0_) versus bending radius, showing similar behavior in all three substrate materials under tension (green) and compression (purple).

Since the bend region decreased in size with smaller bend radii, a geometry correction factor was applied to the measured data by treating the bent and unbent regions as a series of resistors, where the change in resistance is captured entire by the bend region (marked in red, Figure 3B). The normalized resistance was then calculated using eq 1, where the *R*_0_ value is corrected by the bend length for each condition. Figure 3C shows the results generated by static bending across the range of bending radii for all three systems in both tension and compression.

The observed increase in piezoresistive response as a function of bend radius for the specimens oriented in tension aligned well with the response predicted by mechanics for a surface film under tensile bending strain in which plane strain scales as the inverse of bend radius (1/*R*) and the monotonic strain response in Figure 2.^39^ Previous studies of bending in thin film conductors on compliant substrates, observed similar piezoresistive mechanisms, e.g. crack formation, in both monotonic tension and tensile bending indicating that mechanisms observed in the following sections for tensile strain are likely shared with the bending specimens in this experiment.^37,40–42^ In compression, a small decrease in Δ*R*/*R*_0_ values was observed in some conditions which may be explained by increased particle–particle contact or decreased particle–particle spacing as the particle network was compressed. A repeated measurement of the specimen after compressive bending showed a nearly identical value to the initial flat *R*_0_ measurement, indicating that this small decrease in Δ*R*/*R*_0_ is not a permanent change in film conductivity. From an applications perspective, the measured changes in resistance due to bending are not expected to compromise functionality for many active and passive technologies, such as electrodynamic dust shielding and static charge dissipation, indicating that this system may be a good candidate for integrating these technologies into inflatable, flexible, and foldable surfaces.

### 3.3 In Situ Microscopy and Crack Modeling

To understand the contribution of defect formation on the nonlinear electromechanical responses displayed in Figure 2, SEM and optical profilometry were used to assess cracks in the SLNC layer on specimens held at fixed strain values using a custom microtensile fixture. Figure 4 shows SEM images of a HDPE SLNC held at 0, 6, 13, 16, 23, and 35% strain. At 0% strain (Figure 4A) no surface defects were observed, and the SLNC surface appeared smooth and uniform. The light gray speckle pattern was attributed to small variations in surface conductivity due to the composite structure of the SLNC layer which produced contrast in backscatter SEM images. In the HDPE system, cracks in the SLNC layer perpendicular to the strain direction were first observed at 6% strain (Figure 4B), near the point where the electromechanical response began to diverge from the Poisson’s thinning prediction, indicating that crack formation was a primary contributor to the nonlinearity observed at increased strains. The formation of microcracks has been identified as the primary cause of nonlinearity in electromechanical responses in a variety of thin film systems on compliant substrates including, particle mats, embedded particle networks, Ag flake inks, metallic films, and ceramic oxide films.^24,35,42–45^ At higher strains (Figure 4C–E), the crack network evolved via crack lengthening and nucleation of new cracks as indicated by the appearance of small, narrow cracks between wider, longer cracks. This type of network crack evolution is typically observed in piezoresistive systems with high stretchability and moderate gauge factors (10s–100s); in contrast, channel cracking tends to occur in systems with lower stretchability and higher gauge factors (1000s).^45^

**Figure 4:**
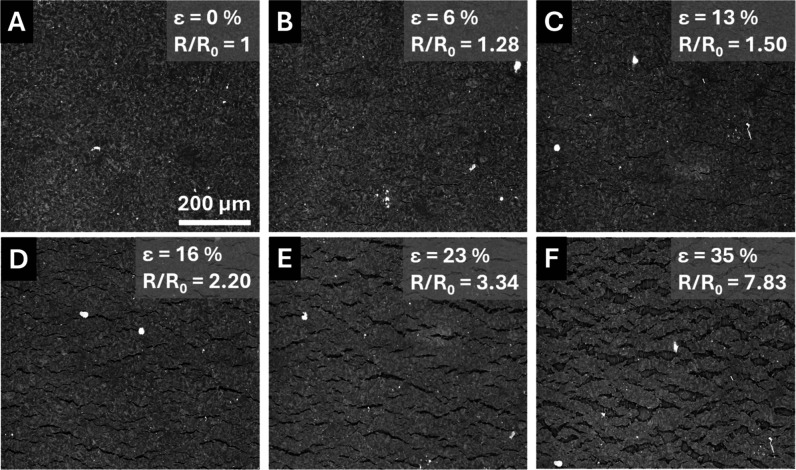
SEM images of HDPE SLNCs at 0, 6, 13, 16, 23, and 35% strain (A–F) showing the crack network evolution at several strain levels and their associated *R*/*R*_0_ values. Images were collected at 500× magnification in BSE mode.

At 35% strain (Figure 4F), significant crack merging resulted in “islands” of SLNC material surrounded by wide cracks exposing bare polymer, as indicated by the strand-like features and charging of these regions during imaging. These SLNC “islands” are connected to neighboring “islands” by bridges of SLNC material producing the high gauge factor response and continued electrical connection at large plastic strains.^45^ Higher magnification images showing a narrower field of view and additional detail for all panels in Figure 4 are included in Figure S4. A secondary electron image is shown in Figure S5 to clearly demonstrate charging of the exposed polymer region noted in Figure 4F. From the images in Figures 4F, S4F, and S5, it is clear that the “islands” of SLNC are still well-adhered to the underlying matrix and do not flake or peel away from the surface, reinforcing the finding that the infiltration process produced well-adhered SLNC films. Additionally, after testing, the cracked SLNC layer could not be removed by scraping or tape lift, which is a significant benefit over ceramic and metallic thin films and unfilled particle films which can generate loose conductive debris during delamination events.^15,46,47^ In low gravity space environments, conductive debris poses a serious risk as the small particles can settle onto critical electronics or lenses, disrupting their functionality.^13^

In the PVDF SLNC system, the initial film morphology (Figure 5A) appeared similar to that of HDPE with slight variations in greyscale due to slight variations in surface conductivity. In PVDF, the initial appearance of cracks also occurred near the crossover point from linear to nonlinear electromechanical behavior (2–3% strain) (Figures 5B and S6B) and lengthened with increasing strain. Figure S6 includes higher magnifications at each strain level in which fine cracks at low strains are easier to resolve. Similar to HDPE, new cracks nucleated between existing cracks as strain increased. By 16% strain (Figure 5E), the SLNC layer contained a set of long cracks that were notably straighter and narrower than the cracks observed at the comparable strain in the HDPE system (Figure 4D). The lower ductility in the PVDF system produced crack morphologies more akin to brittle films on compliant substrates than compliant films on compliant substrates.^15,43,46^ The fracture edge of the PVDF SLNC (Figure 5F) revealed brittle fracture of the SLNC layer, which appeared to be caused by the fusion of a few well-aligned cracks. Beneath the fractured edge of the SLNC, the fracture edge of the PVDF substrate showed a more ductile fracture. Crack fusion along a significant width of the specimen produced a notch-like effect through the SLNC layer, creating a stress concentration leading to early fracture of PVDF substrate at strains well below the fracture of an uncoated PVDF substrate. The notching effect of cracks in the SLNC layer also contributed to the lower strain at break observed in HDPE and OBC SLNC systems, as compared to the bare substrates.

**Figure 5:**
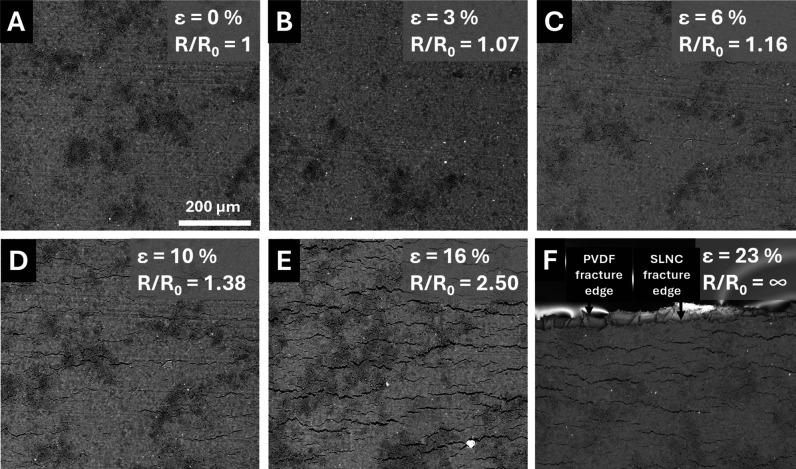
SEM images of PVDF SLNC at 0, 3, 6, 10, and 16% strain (A–E) showing the evolution of the crack network as a function of strain. (F) SEM image of the fracture edge showing brittle fracture of SLNC and ductile fracture of underlying substrate. Images were collected at 500× magnification in BSE mode.

The crack pattern development in the OBC system (Figure 6, and higher magnification images in Figure S7) was most similar to the HDPE systems with a network of tortuous cracks which developed with increasing strain. Interestingly, no cracks were observed at 6% strain, the point at which the electromechanical response became nonlinear, perhaps indicating that the higher ductility of the OBC allowed for a greater contribution of particle network rearrangement to the electromechanical behavior than was observed in HDPE and PVDF.

**Figure 6:**
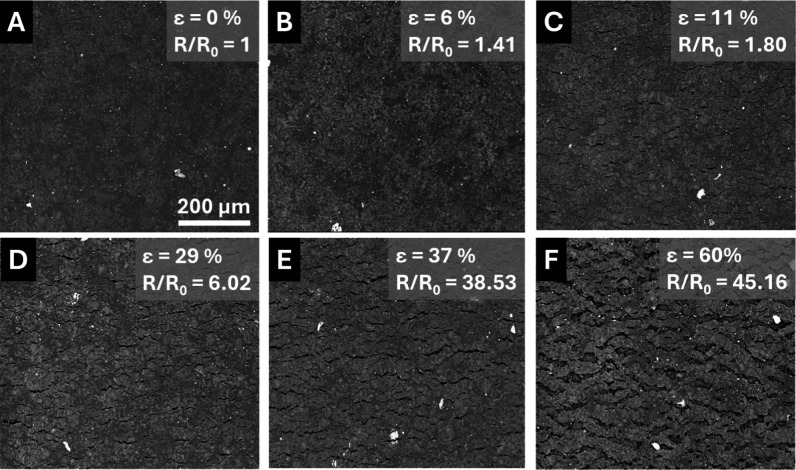
SEM images of OBC SLNCs at 0, 6, 11, 29, 37, and 60% strain (A–F) showing the generation of similar crack patterns as HDPE, as well the formation of SLNC “islands” at high strain values. Images were collected at 500× magnification in BSE mode.

Similar to metallic, ceramic, and other particle films on compliant substrates, crack formation was found to be a common mechanism for nonlinearity in the electromechanical response for all three SLNC systems. The overall crack geometry and crack growth pattern also appeared to align with theoretical and experimental observations on crack network growth with the least ductile system (PVDF) showing the narrowest, longest, straightest cracks, and the most ductile (OBC) showing the shortest, most tortuous crack paths. The higher ductility of HDPE and OBC systems also allowed these systems to support wide crack openings and accounted for the higher stable *R*/*R*_0_ values measured prior to electrical failure. For example, at 60% strain (Figure 6F), the OBC SLNC exhibited a *R*/*R*_0_ of 45, over an order of magnitude higher than the maximum value recorded in the PVDF SLNC system before substrate failure and electrical disconnection.

In order to understand if the observed crack network formation and evolution fully accounted for the nonlinear response in these materials, a crack model was used to predict the expected *R*/*R*_0_ values of the specimens based on the observed crack patterns.^42^ The applied crack model was developed for normalized resistance increases in metallic thin films on polymer substrates.^42^ The model construction assumed that the conductive layer behaved as a uniform 3D conductor with a known length (*l*) and width (*w*) which contained a set of full thickness cracks. The cracks were assumed to be perpendicular to the strain direction, sufficiently smaller than the sample width, and completely nonconductive across the crack face.^42^ Based on these assumptions, a relationship between normalized resistance (*R*/*R*_0_) and linear crack density is described by eqs 3 and 4

3 $$C_{l}=\frac{{Nl}_{0}}{LW}$$

4 $$\frac{R}{R_{0}}=1+\frac{1}{\sqrt{2}}C_{l}{\;l}_{0}+\frac{1}{2}C_{l}^{2}l_{0}^{2}$$

where *N* is the number of cracks, *C*_l_ is the linear crack density, and *l*_0_ is the crack length. Crack lengths were measured by constructing a linear projection of each crack perpendicular the applied strain direction from the SEM images of each SLNC system. An example of the crack segments identified for the PVDF data set are presented in Figure S8. The image magnification used for fitting for each system was selected to limit the population of cracks that extended outside the region of interest. For the PVDF and OBC systems, the higher magnification SEM images in Figures S6 and S7, respectively, were used while the lower magnification images of HDPE SLNCs (Figure 4) were used. For each image, and associated strain step, the linear crack density was calculated using the number of cracks in the image and the image size via eq 3. Since these systems contained a range of crack lengths, the average crack length was used as the *l*_0_ value in the normalized resistance calculation via eq 4. The measured and predicted normalized resistance value for each system are plotted in Figure 7, and presented in Table S3. The relative error, calculated as the difference between the measured and modeled value normalized by the modeled values, is also reported in Table S3 to highlight deviation from predicted values.

**Figure 7:**
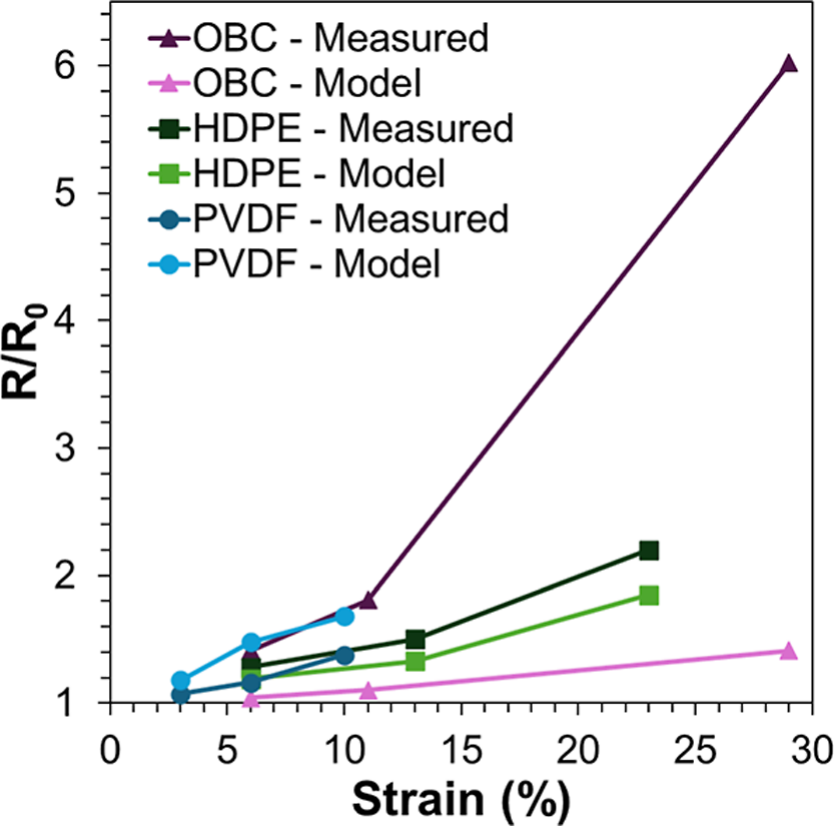
Measured versus modeled normalized resistance values for OBC, HPDE, and PVDF SLNCs.

For the HDPE and PVDF systems, the predicted normalized resistance values were well correlated with the measured values indicating that crack network formation was the primary factor in the nonlinear piezoresistive response for strains beyond yield observed in Figure 2. Additionally, in the case of PVDF, the modeled values were slightly higher than the measured values. This behavior may be attributed to rGO platelets bridging the narrow cracks present in the PVDF system. The large platelet diameter, of a few microns, could allow some platelets to span narrow portions of cracks resulting in an underestimation of resistance. The dramatic deviation from modeled values in the OBC system may be due to the tendency of this system to form localized necking during extension which results in uneven crack distribution. It is possible that the imaged region was not the most deformed region in the sample resulting in a severe underestimation of resistance increase particularly at higher strains.

A complicating factor in the application of models developed for thin metallic or ceramic film systems is the tendency of much thicker nanocomposite films, formed by both ink deposition and the method used in this work, to form partial depth cracks which can grow in both length and depth with increasing monotonic strain or cyclic strain.^24,25^ A partial depth crack would invalidate the assumption of zero-conduction between crack faces and lead to lower measured values than predicted. This reduced effect of crack generation may obscure the contribution of detrimental particle network rearrangement to the piezoresistive response. Optical profilometry was used to measure the crack depth profile at multiple strain values in the HDPE SLNC system. The HDPE system was selected since it represented an intermediate case in piezoresistive properties, observed crack width, and model agreement between the three systems. A confocal digital microscope was used to generate 3D surface profile maps and line cut profiles (Figure 8) of specimens held in fixed strain positions using the microtensile jig described above. Two strain conditions were selected to show the growth of crack depth with increasing strain and the presence of partial depth cracks at strains well beyond yield.

**Figure 8:**
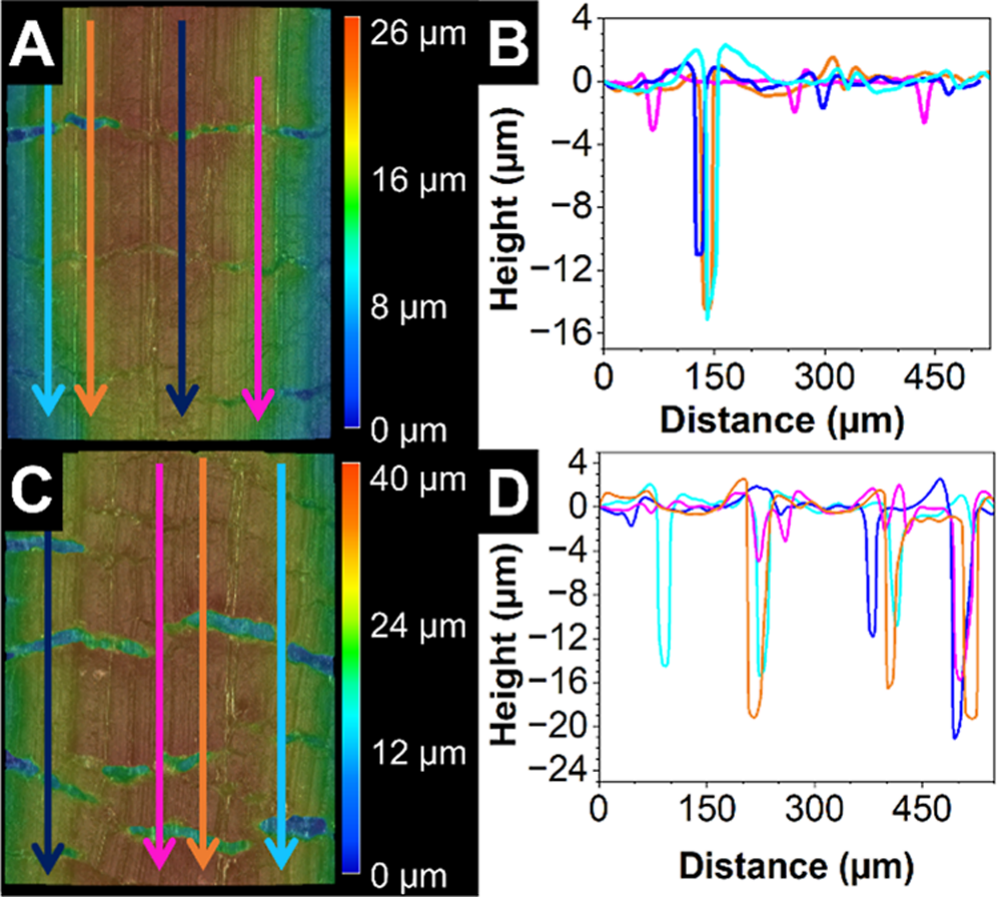
(A) 3D profile map of an HDPE SLNC surface at 18% strain, with arrows indicating the direction of the line cut showing in (B). (B) Line cut profile data showing partial depth cracks. (C) 3D profile map of HDPE SLNC at 35% strain, with arrows indicating the direction of line cut data in (D). (D) Line cut profiles showing full-depth cracks at 35% strain.

In the 18% strain condition, the line cut profiles (marked with colored arrows in Figure 8A) revealed partial thickness cracks which ranged in depth from 3 to 16 μm; less than the 20 μm thickness of the SLNC layer. The presence of partial thickness cracks at strains similar to those examined in the crack modeling experiment (Figure 7 and Table S3) suggest that network rearrangement effects, which increase particle–particle spacing resulting in higher resistivity, may play a greater role in the electromechanical response of the HDPE SLNC than predicted by the small modeling error. Further straining the sample to 35% led to general widening of cracks and deepening of cracks, as observed in the map and line cut profiles in Figure 8D. At this strain value, the 20 μm crack depth and flat peak shapes indicated that a portion of the cracks had propagated through the SLNC to the HDPE substrate.^30^ This mixed mode crack network evolution mechanism is also observed in thick Ag flake filled composite flexible conductors.^24,25^

### 3.4 Cyclic Electromechanical Results

The electromechanical behavior of these materials under cyclic strain conditions was used to better understand how crack network evolution and polymer matrix relaxation influenced the piezoresistive response under cyclic strain conditions which are expected to occur in many potential applications. Figure 9 shows the results of cyclic strain testing in the OBC, HDPE, and PVDF systems at two strain ranges for each system. The lower strain range (left column) represents cycling in the elastic regime while the higher strain range (right column) represents a strain range within the plastic regime, but well before tensile or electrical failure occurs. In each plot, the normalized change in resistance (Δ*R*/*R*_0_) is shown in red, while the measured stress response is shown in black.

**Figure 9:**
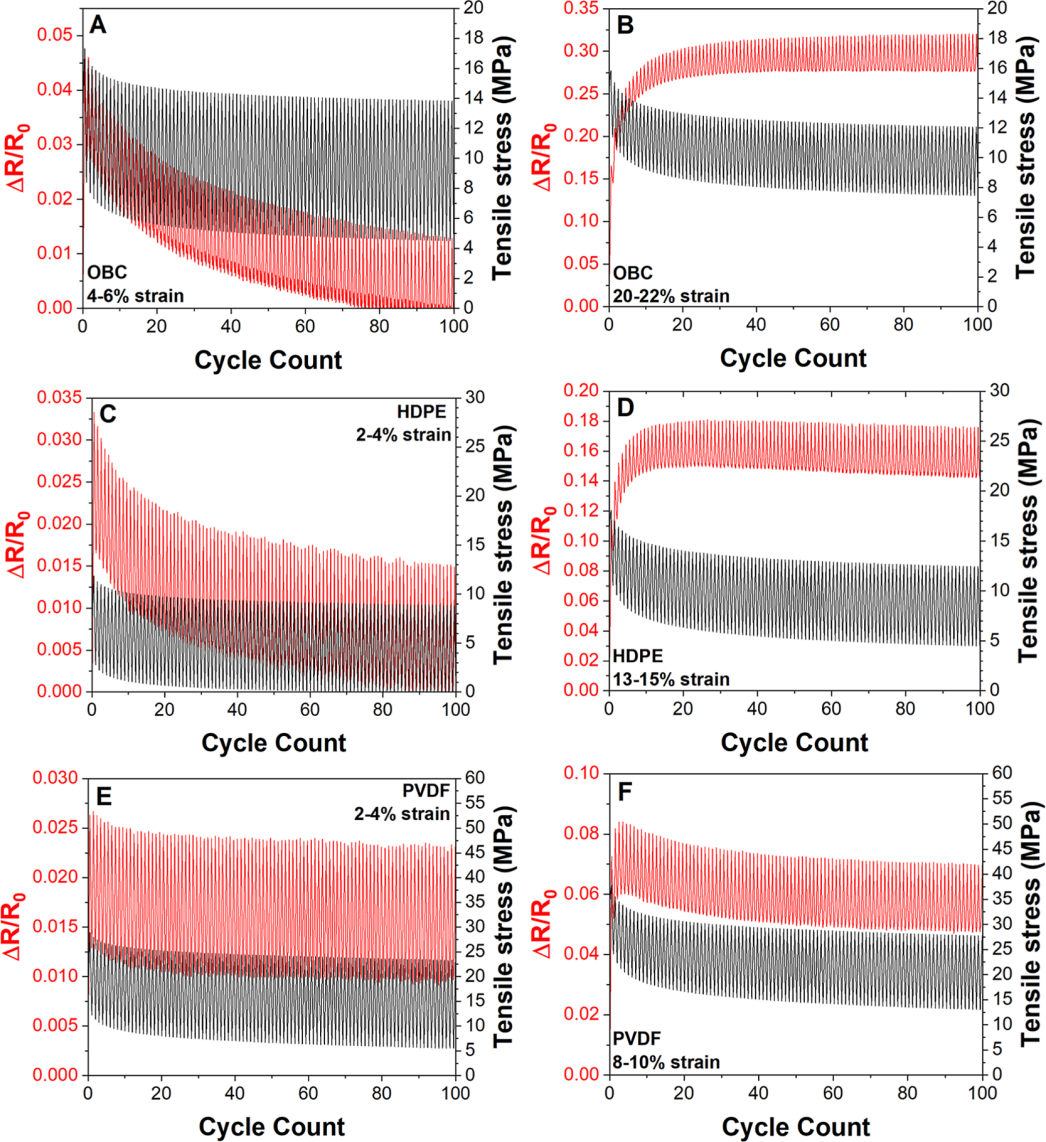
Plots of the normalized change in resistance (Δ*R*/*R*_0_) values (red) and tensile stress response (black) for (A,B) OBC SLNC, (C,D) HDPE SLNC, (E,F) PVDF SLNC showing relaxation of the substrate under all test conditions, but varying piezoresistive responses to relaxation behavior in the low (left column) and high (right column) strain conditions for each substrate.

For the lower strain cycle (Figure 9A,C,E), all systems first exhibited a sharp rise in the minimum and maximum normalized resistance values. This initial increase in both maxima and minima followed by a gradual decay with increasing cycle numbers is correlated with the creep decay of minimum and maximum stress values in the mechanical response. In metallic thin films, such decay behavior has been attributed to fatigue stress induced grain growth, which enhances conductivity, resulting in lower Δ*R*/*R*_0_ minima and maxima.^37^ In nanocomposite systems, enhanced conductivity could be attributed to improved particle network connectivity. This improved connectivity may be driven by changes in particle–particle spacing or particle alignment due to creep behavior.^37^ In semicrystalline polymers subjected to cyclic strain below yield, creep behavior can be attributed to damage events such as nanocavitation and damage of the crystalline lamellae which may allow for beneficial changes in particle–particle positioning and spacing and reduction of resistance.^48^

Additional evidence for the contribution of particle network rearrangement, particularly in the OBC and HDPE systems (Figure 9A,C), is the decay of Δ*R*/*R*_0_ values below the initial measured Δ*R*/*R*_0_ value. Δ*R*/*R*_0_ values below zero cannot occur without some level of beneficial network reconfiguration resulting in increased conductivity of the specimens. In contrast, PVDF SLNCs exhibited a similar decay behavior, but minimum Δ*R*/*R*_0_ values remained above zero. This difference in behavior may be attributed to a small population of cracks visible at 3% strain in this system (Figure 5B) which do not fully close during the lower strain condition contributing to an additional resistivity increase not observed in the HDPE and OBC systems at low strains.

Under high cyclic strain conditions, at strains which resulted in crack network generation during monotonic testing, all three systems showed an initial rise and plateau of Δ*R*/*R*_0_ maxima and minima associated with crack network generation and evolution.^24,37^ In cyclic testing of gold films on polyimide, the initial increase in maximum resistance values has been attributed to the initiation of cracks and the onset of crack propagation. During each cycle, cracks form at energetically favorable positions and begin to propagate resulting in increased maximum resistance with each cycle. Over repeated cycling, damage saturations will occur leading to a plateau of the maxima values.^37^ In the OBC and HDPE systems, this is likely the main contributing mechanism to piezoresistive cyclic response in the plastic regime as a large population of cracks were observed at these strain values in monotonic strain testing (Figures 6D and 4, respectively). The measured magnitude of Δ*R*/*R*_0_ decay attributed to beneficial network rearrangement in these systems (Figure 9A,C) is an order of magnitude smaller than the Δ*R*/*R*_0_ effect attributed to crack network formation and evolution; thus if both mechanisms are occurring simultaneously, the beneficial effects are likely lost in the response signal.

A significantly different behavior was observed in the PVDF system at plastic cyclic strain values (Figure 9F). In PVDF, the piezoresistive response appeared to be a combination of the Δ*R*/*R*_0_ decay behavior attributed to beneficial rearrangement and the rise and plateau behavior attributed to crack network development and growth. This difference in behavior can be attributed to the small overall piezoresistive increase due to crack network generation in the PVDF system compared to the HDPE and OBC systems. The magnitude of recovery attributed to creep mechanisms at lower strains was similar to the overall Δ*R*/*R*_0_ magnitude at higher strains, possibly resulting in the ability to resolve both contributions.

In addition to crack generation, cyclic plastic strains in semicrystalline polymers can also produce a wide variety of longer length scale damage mechanisms including lameller fragmentation, microfibrillation, and microcavity growth and consolidation which may result in severe disruptions in the conductive network. This type of internal damage to the polymer matrix within the composite layer has been directly observed in cyclic deformation of Ag flake based inks.^24^

The Δ*R*/*R*_0_ peak shape can also provide information about crack closure and crack depth in microcracked systems.^37^ In metallic thin film systems, a sinusoidal peak shape during each cycle indicated partial thickness cracking and necking of conductive pathways.^37^ As the cracks propagated to full depth and crack faces experienced complete separation during the strain cycle, the peak shape was observed to transition to a square waveform.^37^ In the high cyclic strain condition (Figure 9B,D,F), the Δ*R*/*R*_0_ response remained sinusoidal for all cycles in all three SLNC systems, suggesting that a portion of the crack population which contributed to the Δ*R*/*R*_0_ response did not propagate through the full SLNC thickness. These observations were consistent with the variation in crack depth measured at a similar strains in for the HDPE presented in Figure 8.

## 4 Conclusions

This study investigated the electromechanical properties of SLNCs in OBC, HDPE, and PVDF systems under monotonic tension, static bending, and cycling loading conditions. Electromechanical measurements made under monotonic tension revealed similar overall piezoresistive behavior across all three systems with variation in magnitude associated with extensibility of the matrix material. Resistance increased gradually in the elastic regime for the OBC and PVDF systems and decreased in the HDPE system, indicating the possibility of particle network rearrangement resulting in reduced resistivity. Beyond yield, all systems experienced a rapid, nonlinear rise in resistance associated with the formation of crack networks in the conductive layer. The extent of strain and maximum measured gauge factor varied with matrix ductility. The more ductile OBC and HPDE systems supported higher gauge factors (20–100+), whereas PVDF only showed a gauge factor of 2–10 before electrical and mechanical failure. The high ductility and corresponding gauge factor sensitivity of the HDPE and OBC systems under tensile strains suggested potential use in detecting high-damage events, such as micrometeoroid impact and puncture events. Static bending results showed electromechanical behavior consistent with basic mechanical model predictions, scaling inversely with bending radius under tension. However, maximum resistance increases at the most severe case tested here (1 mm radius) were modest (Δ*R*/*R*_0_ < 1.2) indicating the suitability of these materials as bendable conductors for both active device and passive applications, such as static charge dissipative surfaces, electrodynamic dust shields, inflatable structures, drapeable covers, and flexible robotics.

In situ optical and scanning electron microscopy revealed that the nonlinear electromechanical response beyond yield was primarily driven by crack formation perpendicular to the stress direction, as is commonly observed for stiff films on compliant substrates. Model fitting of the observed crack patterns indicated that the piezoresistive increase in HDPE and PVDF systems was aligned with the predicted contributions from the crack network. Variation between measured values and predicted values could be attributed to effects such as partial crack bridging by the platelet shaped rGO particles and partial thickness cracks. Poor fitting of the OBC system was attributed to localized necking producing irregular crack distributions along the sample. Optical profilometry in the HDPE system confirmed that partial thickness cracks were formed during tensile strain and that crack network evolution occurred by both crack extension and crack deepening. Cyclic testing in the plastic deformation regime supported the findings that crack network formation and propagation dominated the nonlinear piezoresistive response of these materials. The correlation between the mechanical relaxation of the specimens under cyclic testing in the elastic regime and the piezoresistive response suggested that polymer matrix relaxation effects, which can induce particle network rearrangement, played a significant role in the piezoresistive decay behavior.

Future work on SLNCs in this application space should explore the influence of environmental factors, such as the presence of fine lunar or Martian dust on the piezoresistive response, particularly in cyclic loading where dust may inhibit crack closure effects.^49^ The jagged shape and small size of lunar dust particles may allow them to embed in surface cracks, potentially amplifying resistance changes during cyclic loading of exterior surfaces in lunar exploration scenarios. In addition to application specific testing, future work will include measurement of the mechanical properties of the SLNC layer to provide further insight into the driving forces for crack extension and crack network evolution.

## Supplementary Material



## Notes

Links to GitHub repositories with STL files for the microtensile fixture and the bending jigs. Complete reaction details for rGO functionalization. Additional processing details for SLNC bilayer composite fabrication. DSC thermograms and tabulated data for melt temperature calculation. Electrical measurements of SLNCs. Piezoresistive response plots marked with alternative Poisson’s ratios. Higher magnification SEM images of crack surfaces. Composite image showing crack projection method for crack model fitting. Tabulated data for measured and predicted resistance values of the SLNCs (PDF)

## §

Boeing Aerospace, 7755 E. Marginal Way S., Seattle, WA, 98108, United States

## Abbreviations

SLNC: surface-localized nanocomposite
CMrGO: chemically modified reduced graphene oxide
OBC: olefin block copolymer
HDPE: high density polyethylene
PVDF: poly­(vinylidene fluoride)
rGO: reduced graphene oxide
DSC: differential scanning calorimetry
STL: standard triangle language
ASTM: American Society for Testing and Materials
SEM: scanning electron microscopy
