# Supplementary material for: Origins of Electromechanical Behavior in Surface-Localized Nanocomposites: Insights into Crack Network Dynamics and Particle Network Rearrangements
Source: ACS Appl Polym Mater. 2025 Jul 10;7(14):9211–23. doi: 10.1021/acsapm.5c01519 (PMC12305486; doi:10.1021/acsapm.5c01519)
Supplement: Supplementary file 1 [file ap5c01519_si_001.pdf]

Supporting Information

Origins of Electromechanical Behavior in Surface-Localized Nanocomposites: Insights into Crack Network Dynamics and Particle Network Rearrangements

Authors: Emily A. Ryan [a], Natalie E. Raia [a], John R. Reynolds [a,b], Meisha L. Shofner [a]

[a] School of Materials Science and Engineering, Georgia Institute of Technology, [b] School of Chemistry and Biochemistry, Georgia Institute of Technology

E-mail: meisha.shofner@mse.gatech.edu

Additional file(s): STL files of bending jigs can be found at:

STL files for the micro-tensile fixture can be found at: STL files and usage instructions for the bending jigs and micro-tensile fixture can be found at: https://github.com/mlyryan/Origins_of_Electromechanical_Behavior_Pub or at the digital object identifier: https://doi.org/10.5281/zenodo.15237106.

Synthesis of chemically modified reduced graphene oxide

The chemically modified reduced graphene oxide (CMrGO) used in this study was prepared according to Seibers *et al.* using a 10-day reaction time at a 5g scale.[1] rGO particles (ACS Materials) were heated under vacuum at 110 °C for 24 hours to remove residual water. Dried rGO and a dried polytetrafluoroethylene (PTFE) coated stir bar were placed in a dry, 2L two-neck round bottom flask and purged with nitrogen to prevent water absorption. 500 mg of sodium hydride (Sigma Aldrich) was added to the rGO powder under inert gas conditions and the flask was sealed with rubber septa. 1.5 L of dry, N-methyl-2-pyrrolidone (Sigma Aldrich) was transferred to the reaction vessel via cannula transfer and then a continuous N_2 purge was established to remove any gaseous products from the sodium hydride and rGO reaction. The NMP-rGO mixture was immersed in a silicon oil bath and brought up to 60 °C while being stirred to help disperse the rGO particles. After 1 hour of equilibration, a 10 g charge of 1-bromododecane (Sigma-Aldrich) was added to the reaction vessel through the rubber septa. After 20 minutes, the N_2 purge was removed

and the round bottom remained sealed by the rubber septa, stirring at 60 °C for 10 days to allow for the 1-bromododecane to react with the rGO.

After the reaction was complete, the round bottom was lifted from the oil bath and allowed to cool for 2 hours. 800 mL of methanol (Fischer Scientific) was added to a 2 L beaker, and half the contents of the reaction vessel were added to the methanol to quench any remaining sodium hydride. This NMP-methanol-rGO mixture was then poured over a 1.2 μm PTFE membrane (Cole-Parmer EW-36229-46) supported on glass frit to recover the functionalized nanoparticles. The quenching and filter step was repeated for the second half of the reaction mixture. After all particles were captured on the filter, the product was rinsed with three 500 mL charges of methanol and two 500 mL charges of ethanol (Sigma Aldrich). The washed powder cake was then placed in a clean glass beaker and gently broken up with a PTFE coated rod to aid in solvent removal. The beaker was covered with punctured aluminum foil to reduce turbulence in the vessel during vacuuming and dried under vacuum at 120 °C for 24 hours to full remove residual NMP, methanol, and ethanol. The resulting product is a fine, light, black powder.

Fabrication of Surface-Localized Nanocomposites (SLNCs)

SLNC films were processed in a flat model configuration shown in **Figure S1**. 10 mm thick, flat aluminum plates were used to support the films and four layers of 25 μm thick aluminum foil was placed on each top and bottom surface to act as mold release film. Steel shim stock with a thickness of 95 μm for HDPE and 75 μm for PVDF and OBC were used to set the thickness and prevent significant spreading or thinning of the films during processing. The assembled mold was processed using similar conditions as our prior work.[2] In short, the stacked mold was placed in a melt press (Carver 4386; Wabash, IN) and the top and bottom platens were brought into contact with the mold faces. The mold was then pre-heated for 15 minutes before pressure was applied. The heated mold was subjected to 2.55 MPa of pressure for 30 minutes then water cooled for 10 minutes under pressure before removing the mold from the press. The treatment temperature was selected for each material according to the process below.

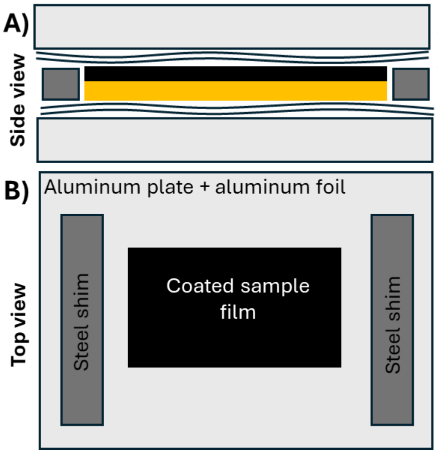

Figure S1. (A) Side view and (B) top view of processing configuration for SLNC fabrication.

SLNC Treatment Temperature Selection

Differential scanning calorimetry (DSC) scans of the as-received poly(vinylidene fluoride) (PVDF) and high density polyethylene (HDPE) films and as-fabricated olefin-block copolymer (OBC) films were collected at a rate of 10 °C/min using aluminum pans and samples masses between 4-6 mg with a DSC Discovery (TA Instruments). The first heating cycle data for each sample is plotted in **Figure S2**. A linear baseline was applied over the plotted data range for each material. A trapezoidal sum integration was applied to the normalized heat flow data (plotted on Y1), and this value was then normalized by the integrated product across the entire plotted region to generate a degree of melt conversion (plotted on Y2). A value of 99.5% melt conversion was selected as the processing temperature for each material as indicated in **Table S1.** The set temperature used on the melt press was set 2 °C higher than the DSC identified temperature to account for thermal lag. The offset value was set to achieve the target temperature within the mold as measured by a K-type thermocouple and Fluke digital thermometer (Fluke, FLK-T3000FC).

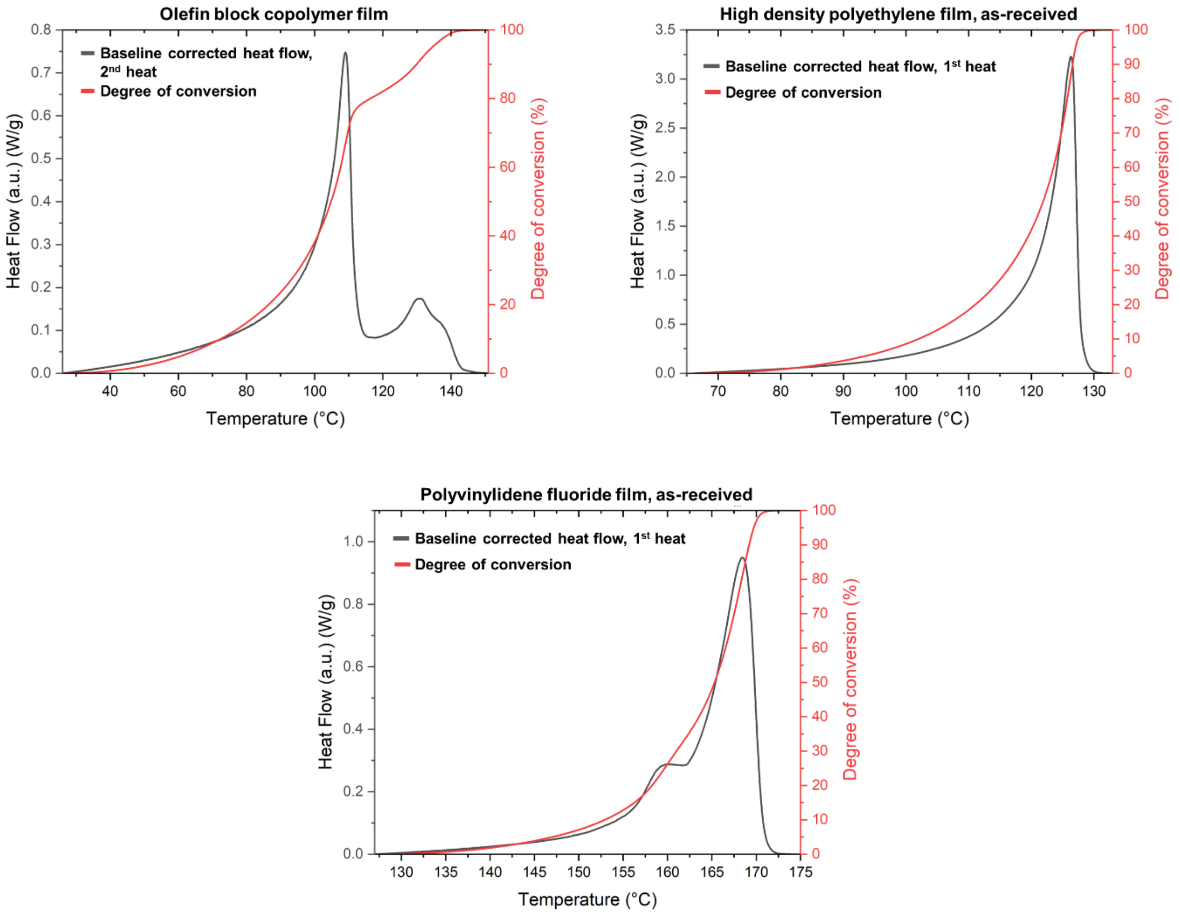

Figure S2. Differential scanning calorimetry (DSC) plots of baseline corrected heat flow versus temperature and associated degree of conversion curves used to select 99.5% melt conversion processing temperatures for each material.

Table S1. Processing temperatures at 99.5% conversion used for infiltration.

**Substrate**	**99.5% Melt (°C)**	**Set Temp (°C)**
OBC	141	143
HDPE	129	130
PVDF	171	173

Table S2. Electrical properties of OBC, HDPE, and PVDF SLNCs as measured by 4pt probe. The mean value and one standard deviation are reported.

**Substrate**	**Sheet resistance** (ohm/sq.)
Particle only on PVDF	265 ± 18
OBC SLNC	321 ± 72
HDPE SLNC	489 ± 134
PVDF SLNC	212 ± 46

Electromechanical properties under monotonic strain

Since the Poisson's ratio of the SLNC materials was not directly measured, **Figure 2** is reproduced with predictions for a wide range of Poisson's ratios typical of polymer and polymer composite materials.

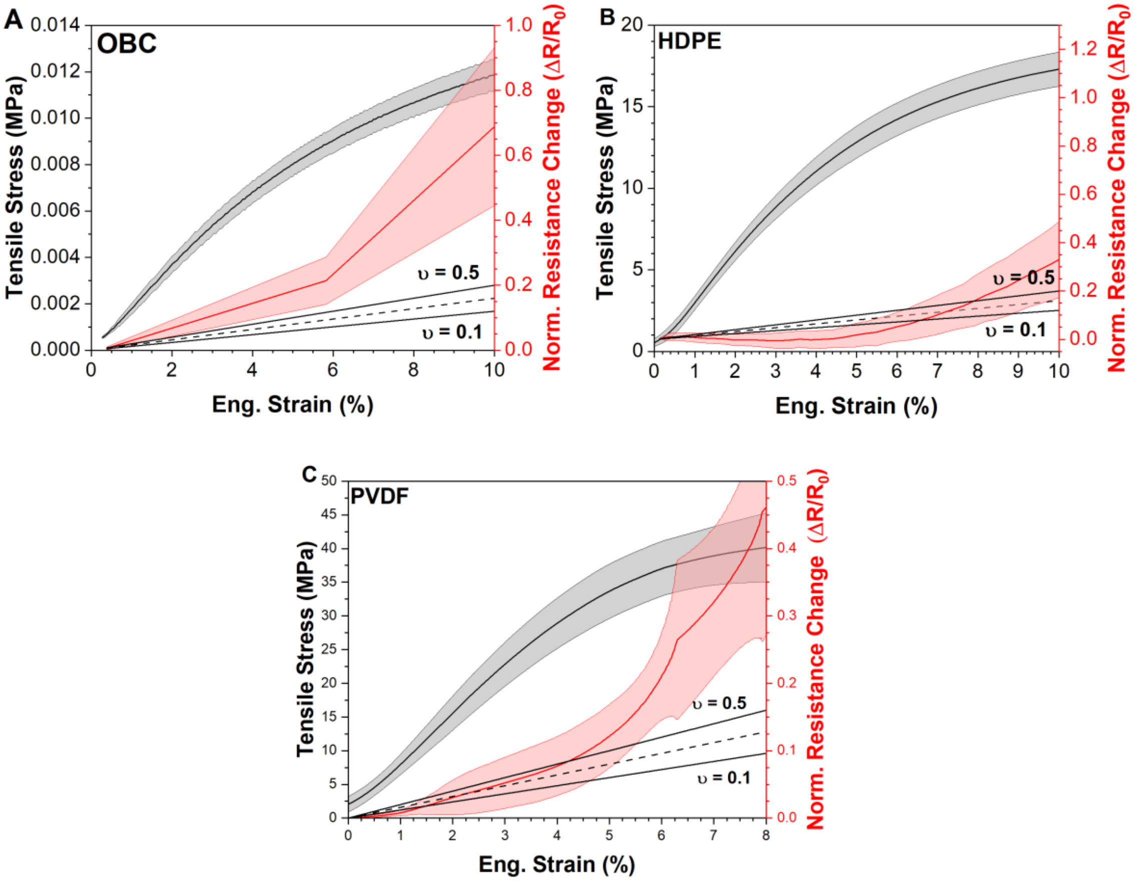

Figure S3. Plots of the piezoresistive response of (A) OBC SLNC, (B) HDPE SLNC, and (C) PVDF SLNCs with lines indicated predicted normalized change in resistance values a piezoresistive response for only Poisson's thinning with Poisson's values of ν=0.1 (solid, bottom), ν=0.3 (dashed, middle), and ν=0.5 (solid, top).

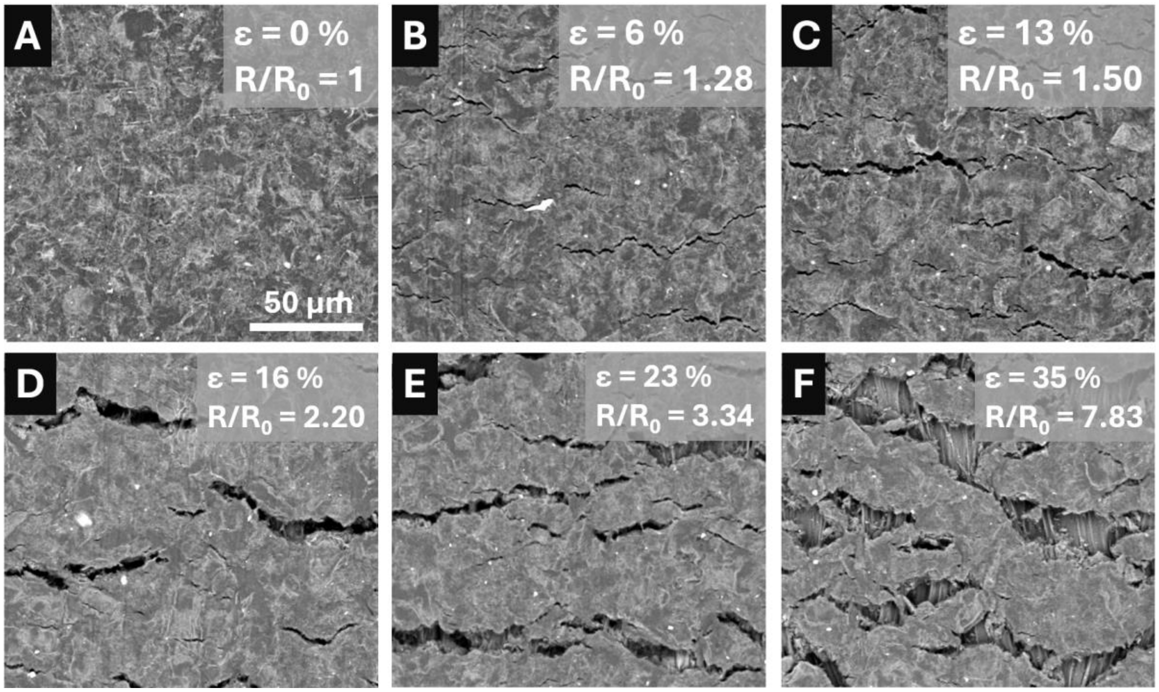

Figure S4. (A-F) Higher magnification (2000x, BSE) SEM images of HDPE SLNCs which highlight the tortuosity of the crack paths and the formation of fibrous features at the bottom of the crack openings at higher strains.

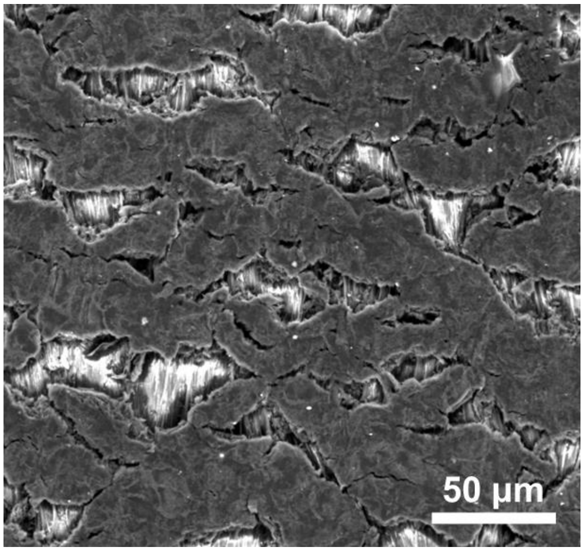

Figure S5. SEM image with similar magnification as **Figure S4F**, collected with the secondary electron detector instead of backscatter to highlight charging (bright white glow) between cracks and at crack edges. No charging is observed in SLNC 'islands' despite the high imaging voltage (10kV) indicating electrical continuity.

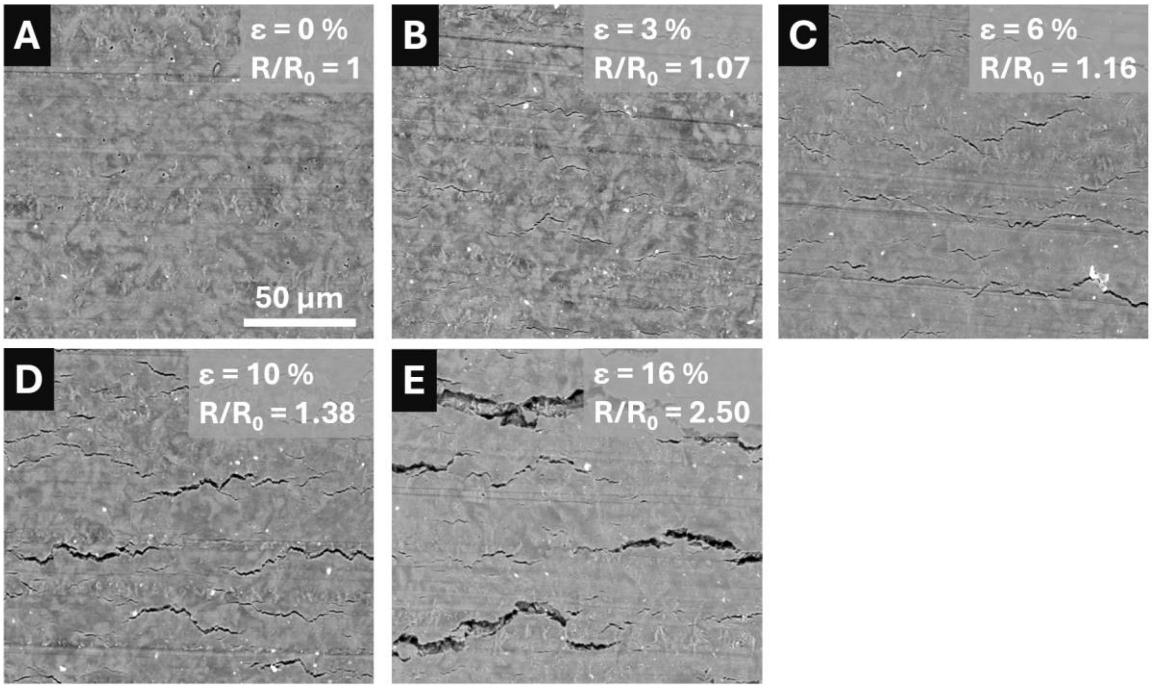

Figure S6. (A-E) Higher magnification SEM images (2000x, BSE) for the PVDF SLNC composite system showing narrower, straighter cracks.

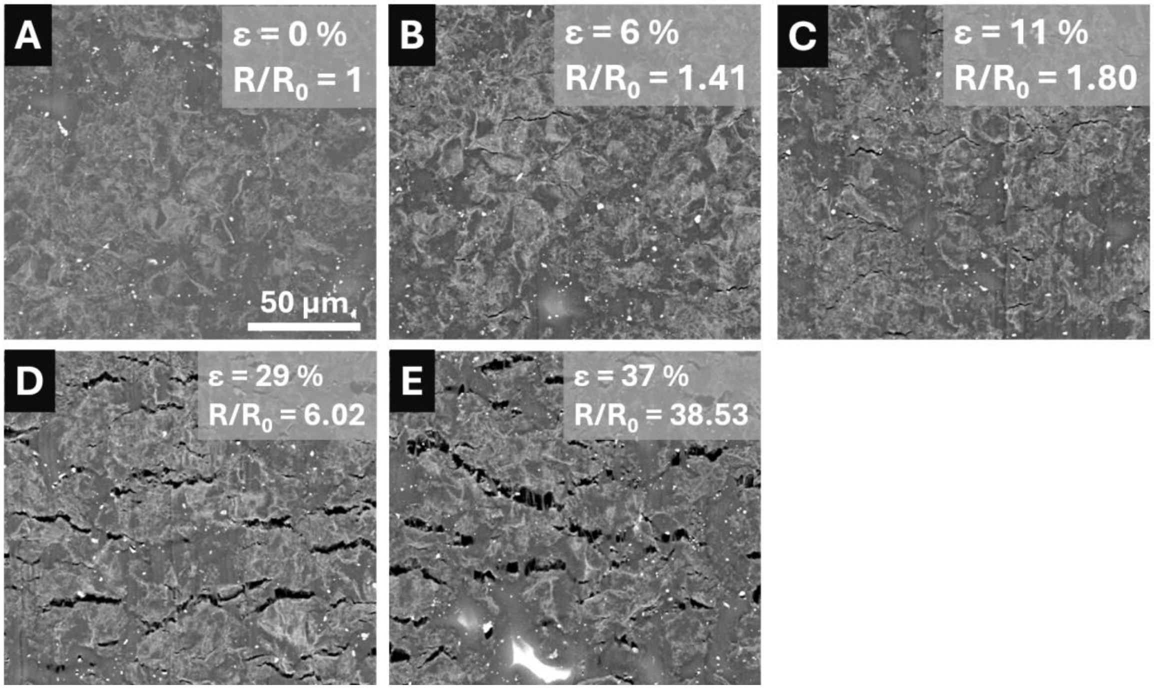

Figure S7. (A-E) Higher magnification SEM images of OBC SLNCs up to 37% strain showing the formation of cracks more similar to the HDPE system. (2000x, BSE)

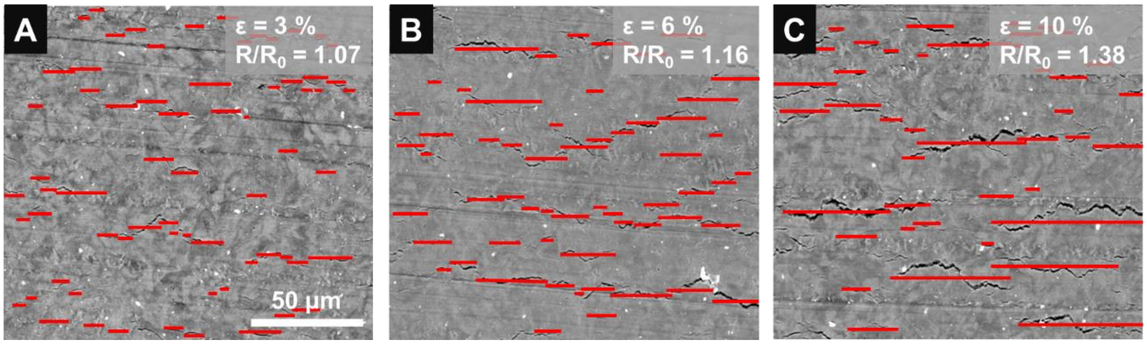

Figure S8. (A-C) Images from high magnification PVDF showing projected crack lengths at three strain points. 0% strain is excluded due to the lack of visible cracks and 16% strain is excluded as crack extend beyond the region of inspection and thus do not meet the assumption that cracks are sufficiently smaller than the sample region.

Table S3. Comparison of actual and predicted normalized resistance values for the SLNCs

**Material – % Strain**	**Number of cracks**	**Crack length (µm)**	**Actual R/R_0 (ohm/ohm)**	**Predicted R/R_0 (ohm/ohm)**	**Relative Error (%)**
PVDF – 3% strain	67	8 ± 4	1.07	1.18	-9%
PVDF – 6% strain	58	13 ± 7	1.16	1.48	-22%
PVDF – 10% strain	43	13 ± 16	1.38	1.68	-18%
HDPE – 6% strain	112	43 ± 20	1.28	1.19	8%
HDPE – 13% strain	137	50 ± 25	1.50	1.33	13%
HDPE – 23% strain	101	68 ± 37	2.20	1.85	19%
OBC – 6% strain	10	12 ± 8	1.41	1.04	35%
OBC – 11% strain	23	12 ± 4	1.81	1.10	64%
OBC – 29% strain	32	18 ± 11	6.02	1.41	327%

References:

(1) Seibers, Z. D.; Brim, E.; Lee Pittelli, S.; Beltran, E.; Shofner, M. L.; Reynolds, J. R. Readily dispersible chemically functionalized reduced graphene oxide nanosheets for solution-processable electrodes and conductive coatings. *ACS Appl. Nano Mater.* **2020**, *3* (11), 11455-11464. DOI: 10.1021/acsanm.0c02539.

(2) Ryan, E. A.; Seibers, Z. D.; Reynolds, J. R.; Shofner, M. L. Surface-localized chemically modified reduced graphene oxide nanocomposites as flexible conductive surfaces for space applications. *ACS Appl. Polym. Mater.* **2023**, *5* (7), 5092−5102. DOI: 10.1021/acsapm.3c00588.
